# Age-related maculopathy and sunlight exposure evaluated by objective measurement

**DOI:** 10.1136/bjo.2007.130575

**Published:** 2008-04-25

**Authors:** M Hirakawa, M Tanaka, Y Tanaka, A Okubo, C Koriyama, M Tsuji, S Akiba, K Miyamoto, G Hillebrand, T Yamashita, T Sakamoto

**Affiliations:** 1 Department of Ophthalmology, Kagoshima University Graduate School of Medical and Dental Sciences, Kagoshima, Japan; 2 Department of Epidemiology and Preventive Medicine, Kagoshima University Graduate School of Medical and Dental Sciences, Kagoshima, Japan; 3 Procter & Gamble, Kobe, Japan

## Abstract

**Aim:**

To study the relationship between age-related maculopathy (ARM) and exposure to sunlight using an objective method.

**Methods:**

In a case–control study of Japanese men aged ⩾50 years (67 controls without ophthalmic disease and 148 with ARM), those with ARM were separated into groups of early (n = 75) and late (n = 73) ARM. Facial wrinkle length and area of hyperpigmentation, which are considered to be associated with exposure to sun, were measured using imaging with computer-based image analysis. Skin tone was also measured on the upper inner arm, which is not exposed to sun. Early and late ARM association with skin measurements was then evaluated.

**Results:**

Significantly more facial wrinkling (p = 0.047, odds ratio 3.8; 95% CI 1.01 to 13.97) and less facial hyperpigmentation (p = 0.035, odds ratio 0.3; 95% CI 0.08 to 0.92) was present in late ARM cases. The relationship between skin tone and ARM risk was not statistically significant.

**Conclusions:**

This objective method showed that lifetime exposure to sunlight is an important factor in the progression of late ARM. An individual’s reaction to sunlight exposure may have a role in ARM progression in addition to total lifetime exposure to sunlight.

The aetiology of age-related maculopathy (ARM), which is the most common cause of vision loss in older people in developed countries, remains unclear, but is suspected to involve both external and internal factors.^1^^–^7 Of the external factors, smoking is the most well-established independent risk factor.^4^^–^7 In contrast, there is controversy over the role of other potential external factors, such as exposure to sunlight or ultraviolet radiation (UV).^8^^–^16 It has been reported that abnormal skin sensitivity to sunlight or a propensity to tan is associated with ARM.^11^^–^13 However, there are several reports that sunlight exposure is not a risk factor related to ARM.^14^^–^17

The controversy is probably due to the methods used to measure lifetime exposure to sunlight. Most studies assessed total sunlight exposure by using questionnaires, and the accuracy of the data obtained depends heavily on question “quality” and respondents’ memory. This is an inevitable and unsolvable problem of questionnaire methodology.^8^^–^17

We previously reported^18^ ^19^ that people with different lifetime exposures to sunlight have correspondingly different severities of facial skin wrinkling and hyperpigmentation. In those earlier studies, we used video imaging combined with image analysis to objectively quantify skin features, reasoning that wrinkling and hyperpigmentation were quantitative, objective biomarkers of the exposure of people of the same gender and ethnic group, and thus measured true lifetime exposure more accurately than questionnaires. We used these measurements to evaluate the relationship between facial wrinkling and hyperpigmentation and ARM.

## SUBJECTS AND METHODS

This case–control study of ARM and healthy controls involved subjects seen at Kagoshima University Hospital or Kagoshima Kouseiren Hospital Health Care Center between May 2005 and February 2006 who met the inclusion criteria below, and were asked to participate after the study was carefully explained. Inclusion criteria were as follows:

1. Life-long residence in Kagoshima prefecture
2. Aged 50 years or older and male
3. Fundus photographs could be taken
4. Ocular fundi were observable
5. Absence of self-reported ocular disease, eg, glaucoma or diabetic retinopathy

Late ARM cases were those diagnosed at Kagoshima University Hospital during the study. Controls and early ARM cases had undergone health checks at Kagoshima Kouseiren Hospital Health Care Center during the same period.

An initial assessment of 259 participants excluded 44: 18 had media opacity and 26 had ocular diseases (four with diabetic retinopathy, one with branch retinal vein occlusion, three with glaucoma, five with epiretinal membrane, and 13 with polypoidal choroidal vasculopathy). The 215 subjects who met the inclusion criteria comprised 67 controls, 75 with early ARM and 73 with late ARM. All subjects with late ARM had neovascular membrane confirmed by angiography. No geographic atrophy was seen.

Our research followed the tenets of the Declaration of Helsinki, with informed consent obtained from the subjects, and was approved by all of the institutional review boards involved.

### Fundus examination

Fundus colour photographs (45°) of the macula (Canon CR-DG10, Tokyo, Japan) were graded by two independent qualified judges (MH, AO), who had no contact with the subjects. ARM was defined on the basis of the International ARM Epidemiological Study Group classification^20^: early ARM by the presence of soft drusen (⩽63 μm) or retinal pigment epithelium pigmentation abnormalities within the grid, and late ARM by either neovascular age-related macular degeneration or geographic atrophy involving the fovea. Minimum geographic atrophy was a circle of 175 μm or more in diameter. Those with fundus inflammatory or retinovascular disease, choroidal neovascularisation due to high myopia, or polypoidal choroidal vasculopathy confirmed by fluorescein and indocyanine green angiography were excluded. Classification was based on the subject’s worst eye.

### Smoking

Smoking history was obtained from questionnaires, with lifetime smoking exposure quantified in “pack-years”, one “pack year” being 20 cigarettes smoked per day for one year.^21^

### Hypertension

Blood pressure was measured three times with the subject in a sitting position, and the mean was used for analysis. Hypertension was defined as systolic blood pressure ⩾140 mm Hg, diastolic blood pressure ⩾90 mm Hg, or current use of antihypertensive drugs.

### Skin examination

#### Wrinkles

**Figure 1:**
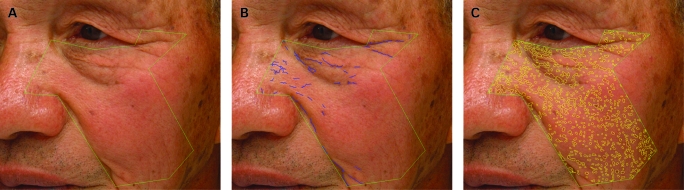
Representative images used to quantify facial wrinkling and hyperpigmentation. (A) The region of interest (ROI) was demarcated manually as shown by the green line. (B) The facial wrinkles detected in the ROI are shown (blue lines). (C) The hyperpigmented regions detected in the ROI are shown (yellow). Patient consent has been obtained for publication of this figure.

The total length of facial wrinkles in the region of the upper cheek and temporal areas next to the eyes was objectively measured using a two-dimensional imaging system using a commercially available high-resolution digital camera equipped with a close-up lens mounted in a standardised illumination box fitted with head-positioning aids (Beauty Imaging System; Procter & Gamble, Cincinnati, Ohio, USA). The camera was calibrated daily using a GretagMacbeth neutral 8.0 grey colour board in front of the camera. Left and right views of the face were standardised—that is, the same focal distance from the camera lens to the face, same magnification, same head position so that the camera angle was the same relative to the face surface, and exactly the same lighting.^18^ ^19^ ^22^ ^23^ The region of interest (ROI) was marked manually based on 12 predefined facial landmarks around the eye and cheek—for example, corners of the eye, bridge of the nose, corners of the mouth (fig 1). The lengths of facial wrinkles (fine lines) in the ROI were quantified objectively using image analysis algorithms based on an Optimus software platform, which automatically locates each facial line and quantifies the total number, length and area of facial lines longer than 5 mm and more than 0.16 mm wide, known magnification used to convert pixel data to actual length and area data. Thresholds were based on “clinically important” wrinkling—that is, excluding lines shorter than 5 mm and narrower than 0.16 mm, which fall under the heading of surface “texture”.

Because the ROI varies in shape and size, total wrinkle area was normalised to total ROI size to yield a wrinkle area fraction (WAF)—that is, fractional ROI area occupied by wrinkles or fine lines. WAF varied from 0.05 (5% of ROI) to 0.2 (20% of ROI) depending on individual severity of wrinkling. Group statistical analysis used the mean WAF on the left and right sides of the face for each subject. The intraindividual coefficient of variation of imaging (within-subject reproducibility) quantifying wrinkling was found previously to be 5.2%.^23^ Accuracy was confirmed using mannequins with artificial wrinkles of known length and width. Imaging accuracy was ±5% of the actual value.^23^

#### Pigmentation

Total facial hyperpigmentation on the left and right sides was objectively measured using the Beauty Imaging System. The region hyperpigmentation was defined as a localised region of darker skin. Hyperpigmentation is often observed after inflammation, melasma and senile lentigines, and can be exacerbated by exposure to sun.^18^ ^19^ The ROI in each image was defined manually and then automatically analysed using customised software that locates and quantifies the total area of hyperpigmented spots. The total area of spots was then normalised to the total area of the region analysed. This analysis was conducted on both the left and right sides of the face, and the mean of the two sides was used as the final measure of hyperpigmentation for each subject in the group statistical analysis.

#### Skin tone

Skin tone was measured on the upper inner arm using a colour reader (CR-13; Minolta, Tokyo, Japan), which was calibrated using the standard white plates supplied with the instrument,^18^ ^19^ ^22^ to obtain three skin tone indices L*, a* and b*, ie, lightness, redness and yellowness. Triplicate measurements at each site were averaged and analysed. Skin on the inner arm represents constitutive skin colour because it is not exposed to sun.

### Sample size

From previous work, the sun sensitivity index odds ratio (OR) for late ARM was 3,^13^ so sample size was determined to detect ORs of 3.0 with 80% statistical power at p⩽0.05%. The required sample size was 66 in each group.

### Statistical analysis

Stata 8.1 (Stata Corp, Lakeway Drive College Station, Texas, USA) was used for the statistical analysis. A multivariate logistic regression analysis obtained the maximum likelihood OR estimates and corresponding 95% CIs using ARM stage as a dependent variable. The possible risk factors, age, smoking and hypertension, and sun-related skin factors, facial wrinkling, facial hyperpigmentation and skin tone L*, a* and b*, were included in the models as covariates. Subjects were separated by age into the following groups: <60, 60–64, 65–69, 70–74 and ⩾75. Smoking history was analysed as non-smoking, 0–24 pack-years and 25 pack-years or more. Hypertension was dichotomised into absence and presence. Each variable of the skin examination, wrinkling, hyperpigmentation and skin tone (L*, a* or b*), was divided into upper, middle and lower tertiles referenced in statistical analyses. Trend was analysed by a likelihood ratio test using categorical data as continuous variables. Two-sided p<0.05 was considered to be significant.

## RESULTS

### Study group baseline

Table 1 lists subjects on the basis of age, smoking status, hypertension, facial wrinkling, facial hyperpigmentation and skin tone.

**Table 1:** Characteristics of study subjects

Characteristic	Control(n = 67)	EarlyARM(n = 75)	LateARM(n = 73)
Age (years)			
<60	25 (37.3)	7 (9.3)	5 (6.8)
60–64	11 (16.4)	10 (13.3)	5 (6.8)
65–69	12 (17.9)	18 (24.0)	14 (19.2)
70–74	17 (25.3)	26 (34.7)	14 (19.2)
⩾75	2 (3.0)	14 (18.7)	35 (47.9)
Smoking (pack-years)			
0	32 (47.8)	37 (49.3)	11 (15.1)
1–24	16 (23.9)	14 (18.7)	25 (34.2)
⩾25	19 (28.4)	24 (32.0)	37 (50.7)
Hypertension			
Absent	35 (52.2)	34 (45.3)	25 (34.2)
Present	32 (47.8)	41 (54.7)	48 (65.8)
Facial wrinkling			
<0.104	32 (47.8)	18 (24.0)	20 (27.4)
0.104–0.1314	20 (29.9)	29 (38.7)	22 (30.1)
⩾0.1314	15 (22.4)	28 (37.3)	31 (42.5)
Facial hyperpigmentation			
<0.0264	19 (28.4)	20 (26.6)	31 (42.5)
0.0264–0.0342	24 (35.8)	27 (36.0)	20 (27.4)
⩾0.0342	24 (35.8)	28 (37.3)	22 (30.1)
Upper inner arm skin tone L*			
<59.7	15 (22.4)	26 (34.7)	28 (38.4)
59.7–62.1	21 (31.3)	30 (40.0)	24 (32.9)
⩾62.1	31 (46.3)	19 (25.3)	21 (28.8)
Upper inner arm skin tone a*			
<7.2	33 (49.3)	21 (28.0)	18 (24.7)
7.2–8.5	21 (31.3)	25 (33.3)	25 (34.2)
⩾8.5	13 (19.4)	29 (38.7)	30 (41.1)
Upper inner arm skin tone b*			
<14.7	29 (43.3)	18 (24.0)	26 (35.6)**?**
14.7–16.7	19 (28.4)	24 (32.0)	24 (32.9)
⩾16.7	19 (28.4)	33 (44.0)	23 (31.5)

Values are number (%).

ARM, age-related maculopathy.

### Age

Mean (SD) age was 63.1 (8.1) years for the controls, 68.7 (6.9) years for the early ARM group, and 72.8 (7.9) years for the late ARM group. The early ARM (p = 0.001) and late ARM (p<0.001) groups were significantly older than the controls. Age was a significant factor related to both types of ARM (table 2).

**Table 2:** Age, smoking status and hypertension in subjects with age-related maculopathy (ARM) compared with controls

Risk factor	Early ARM vscontrols	Late ARM vscontrols
Age (years)		
<60	1	1
60–64	3.7 (1.00 to 13.51)	2.8 (0.45 to 17.80)
65–69	5.3 (1.44 to 19.33)	9.7 (1.94 to 48.89)
70–74	5.7 (1.68 to 19.19)	7.3 (1.52 to 34.84)
⩾75	25.1 (3.70 to 170.49)	114.5 (15.07 to 869.73)
p Value for trend	0.001	<0.001
		
Smoking (pack-years)		
0	1	1
0–25	1.2 (0.40 to 3.55)	5.2 (1.39 to 19.36)
⩾25	0.9 (0.35 to 2.30)	5.4 (1.61 to 17.90)
p Value for trend	0.856	0.007
		
Hypertension		
Absent	1	1
Present	1.1 (0.51 to 2.48)	1.1 (0.41 to 3.18)
p Value	0.783	0.788

Values are OR (95% CI) adjusted for age, smoking, hypertension, facial wrinkling, facial hyperpigmentation and skin of upper inner arm (L*, a* or b*).

### Smoking

Smoking data (mean (SD)) were as follows: 16.1 (21.0) pack-years for controls, 17.8 (25.0) pack-years for the early ARM group, and 32.4 (29.0) pack-years for the late ARM group. Logistic regression analysis showed smoking to be significantly related to late ARM (p for trend  = 0.007), but not early ARM (table 2).

### Hypertension

Hypertension was not a significant risk for ARM groups (table 2).

### Wrinkles

**Figure 2:**
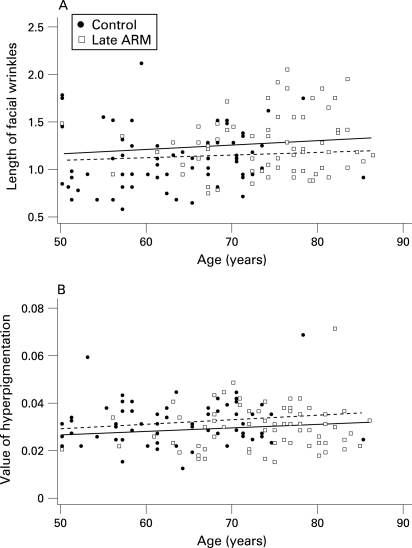
Facial wrinkling (A) and facial pigmentation (B) of patients with late age-related maculopathy (ARM) and controls according to age. (A) Regression lines were drawn separately for patients with late ARM (solid line: y = 0.09168+0.00047x) and controls (dotted line: y = 0.08670+0.00040x). (A) Wrinkle area fraction (WAF) increased with age in patients with late ARM and controls. However, the observed age dependence of WAF was not statistically significant in either patients or controls. (B) Regression lines were drawn separately for patients with late ARM (solid line: y = 0.01898+0.00015x) and controls (dotted line: y = 0.02152+0.00016x). Facial pigmentation increased with age in patients with late ARM and controls. However, the late ARM group was significantly less pigmented than the control group. Data for the early ARM group were not included to avoid overlapping of points.

WAF increased with age in patients with ARM and controls (fig 2). However, the observed age dependence of WAF was not statistically significant in either patients or controls. Logistic analysis showed that patients with late ARM had larger WAFs on average than controls after adjustment for age, smoking, hypertension, facial hyperpigmentation and skin tone (p for trend  =  0.047), but patients with early ARM did not (table 3).

**Table 3:** Wrinkles, pigmentation and skin tone in subjects with age-related maculopathy (ARM) compared with controls

Risk factor	Early ARM vscontrols	Late ARM vscontrols
Facial wrinkling		
<0.104	1	1
0.104–0.1314	1.4 (0.53 to 3.49)	1.9 (0.54 to 6.56)
⩾0.1314	2.5 (0.89 to 7.08)	3.8 (1.01 to 13.97)
p Value for trend	0.086	0.047
		
Facial hyperpigmentation		
<0.0264	1	1
0.0264–0.0342	0.6 (0.24 to 1.69)	0.3 (0.08 to 0.92)
⩾0.0342	0.6 (0.21 to 1.56)	0.3 (0.08 to 0.92)
p Value for trend	0.332	0.035
		
Upper inner arm skin tone L*		
<59.7	1	1
59.7–62.1	0.9 (0.30 to 2.57)	0.5 (0.13 to 2.14)
⩾62.1	0.9 (0.23 to 3.35)	0.6 (0.10 to 2.94)
p Value for trend	0.848	0.492
		
Upper inner arm skin tone a*		
<7.2	1	1
7.2–8.5	1.5 (0.51 to 4.37)	1.5 (0.38 to 5.64)
⩾8.5	2.1 (0.58 to 7.69)	1.8 (0.37 to 9.17)
p Value for trend	0.257	0.120
		
Upper inner arm skin tone b*		
<14.7	1	1
14.7–16.7	1.1 (0.38 to 2.97)	0.7 (0.18 to 2.50)
⩾16.7	1.2 (0.43 to 3.56)	0.3 (0.08 to 1.34)
p Value for trend	0.697	0.120

Values are OR (95% CI) obtained from the logistic model incorporating age, smoking, hypertension, facial wrinkling, facial hyperpigmentation and upper inner arm skin tone (L*, a* and b*).

### Pigmentation

Facial pigmentation increased with age in patients with ARM and controls (fig 2). Here again the observed age dependence was not significant in either patients or controls. Logistic analysis showed that patients with late ARM had smaller areas of pigmentation on average than controls after adjustment for age, smoking, wrinkling and upper inner arm skin (L*, a* and b*). In late ARM cases, a significantly smaller area of hyperpigmentation was found (p for trend  = 0.035, table 3). There was no significant difference in area of hyperpigmentation between patients with early ARM and controls.

### Skin tone

Mean (SD) indices of skin tone (L*, a* and b*) were: 61.9 (2.9), 7.6 (1.6) and 15.4 (2.6), respectively, for controls; 60.4 (2.3), 8.4 (1.9) and 16.3 (2.2), respectively, for early ARM; 60.4 (3.0), 8.3 (1.8) and 15.4 (2.6), respectively, for late ARM. Patients with ARM had darker, redder and more yellow skin, but no significant difference was seen in skin tone (L*, a* and b*) among the groups (table 3).

## DISCUSSION

This paper is, to our knowledge, the first to objectively quantify lifetime exposure to sun and to evaluate the relationship between this and ARM.

Our study shows that facial wrinkle length is positively related to late ARM prevalence, so lifetime exposure to sunlight and late ARM are considered to be positively related. Although it is conventionally held that hyperpigmentation and late ARM are related to lifetime exposure to sunlight, our results, in fact, show the opposite—that is, people with late ARM have fewer facial hyperpigmentation spots. This is difficult to explain; however, a relationship may exist between individual characteristics—that is, a skin that is strongly resistant to tanning and ARM.

Skin wrinkling has been suspected to be strongly associated with lifetime exposure to sunlight.^22^^–^24 Skin wrinkling is related to an increase in collagen degradation in the extracellular matrix and to a decrease in its synthesis.^25^ UV irradiation affects wrinkle formation through a cumulative process, so it is logical that facial wrinkling may be an indicator of lifetime exposure to sunlight.^18^ ^19^ ^22^

A significantly smaller area of hyperpigmented spots was, however, found in late ARM, despite the fact that our previous report concluded that hyperpigmentation was also increased by lifetime exposure to sun.^18^ It may be that sunlight/UV irradiation induces changes in skin pigmentation, but the biological pathway differs from that of wrinkle formation. Skin darkening in response to UV irradiation occurs via two distinct pathways: immediate pigment darkening and delayed tanning. Delayed tanning involves melanogenesis and is stimulated by DNA photodamage or its repair.^26^ Melanin synthesis is therefore thought to be a direct response to DNA damage, and melanogenesis is viewed as a biomarker of DNA repair capacity. People with less pigmentation may be more vulnerable to DNA damage caused by exposure to sunlight. Type 1 Fitzpatrick skin types, those with fair-coloured skin and poor tanning ability, are known to be highly susceptible to skin cancer.^27^ ^28^ We hypothesise that genetic factors related to skin pigmentation are also related to ARM progression in Japanese men in Kagoshima.

With regard to the retina, melanin can act biochemically as an antioxidant in retinal pigment epithelial cells, lessening the harmful effects of UV-induced oxygen free radicals.^28^ Ocular melanin is thought to be able to physically protect the retina and choroid of pigmented eyes against light-induced cell toxicity through UV absorption. The protection against UV damage afforded by melanin, presumably through a biochemical mechanism, may explain why ARM is more common in lighter-skinned populations.^29^

This study has certain limitations. There may be concern that exposure of facial skin to sunlight does not exactly reflect exposure of the ocular fundus to sunlight. All participants were male farmers with similar lifestyles in this rural area—for example, sunglasses are rarely used, diets are similar and individual differences would not be large. Although the sample was not large, two well-known risk factors for ARM, aging and smoking, were found to be significant for ARM. Because greater facial wrinkling is also a significant risk factor for late ARM, the sample size was sufficient for a preliminary identification of an additional risk factor for this condition. A potential subject selection bias exists in the study. Subjects with greater exposure to sunlight tend to have severe cataracts and may have been excluded because fundus photographs could not be taken. Of particular note is that age was not matched equally in the ARM groups compared with controls. Although age was applied as a covariant and the multiple variant analysis was performed in an age-adjusted way, the present results should be interpreted with care. It is not possible to say that the present method is perfectly objective. In fact, the definition of wrinkle that we used is subject to interpretation. Although the algorithm thresholds for wrinkle detection are subject to interpretation, the use of computer image analysis to objectively quantify wrinkle length and areas of hyperpigmentation eliminates the potential for bias and error associated with standard visual grading.

There are various other factors that can affect skin condition. Wrinkle length and/or skin pigmentation does not indicate the risk of ARM in every individual clinically. The value of the present method lies rather in its usefulness to study the pathogenesis of ARM.

In conclusion, this study suggests that lifetime exposure to sunlight is associated with ARM in Japanese men living in Kagoshima, Japan. Individual response to acute/chronic exposure to sun may be important in the progression of ARM. Because the objective methods used to measure skin wrinkling and hyperpigmentation such as markers of lifetime exposure to sunlight are standardised across all subjects in the study, and are cost-effective, reproducible and non-invasive, a large-scale follow-up study on different populations would be warranted to better elucidate the role of sun exposure in the progression of ARM and would be useful in the design and development of effective prophylactic treatments.
